# Comparative entomological study on ecology and behaviour of *Anopheles* mosquitoes in highland and lowland localities of Derashe District, southern Ethiopia

**DOI:** 10.1186/s13071-014-0483-9

**Published:** 2014-10-20

**Authors:** Terefe Gone, Meshesha Balkew, Teshome Gebre-Michael

**Affiliations:** Department of Medical Laboratory Sciences, Hossana College of Health Sciences, P.O. BOX 159, Hossana, Ethiopia; Department of Entomology and Vector Biology, Aklilu Lemma Institute of Pathobiology, Addis Ababa University, Addis Ababa, Ethiopia

## Abstract

**Background:**

Change in climatic and socio-economic situations is paving the way for the spread of malaria in highland areas which were generally known to be malaria free. Despite this, information regarding highland malaria transmission is scarce. Thus, the present study investigated entomological parameters linked to malaria transmission in the highlands of Southern Ethiopia.

**Methods:**

A longitudinal entomological study was conducted in three localities situated at different altitudes ranging between 1300 and 2650m above sea level in Derashe district, Southern Ethiopia. Larval and adult anopheline mosquitoes were collected between October 2011 and February 2012.

**Results:**

*An. arabiensis* and *An. funestus* s.l existed at significantly higher densities in the lowland (Wozeka) in contrast to *An. christyi* and *An. Demeilloni,* which were more abundant in the highland localities (P < 0.01). Conversely, *An. pharoensis* and *An. cinereus* were scarce and only found in the lowland and highlands, respectively. Habitats of larvae of *An. arabiensis* were characterized as clear, sun-lit, permanent, still water (streams) without vegetation and situated close to human habitations. On the other hand, habitats of *An. christyi* are shaded, still, turbid and contain natural water (rain pools) with vegetation and mats of algae. The relative abundance of *An. Arabiensis,* which is the primary malaria vector in Ethiopia is significantly and positively correlated with water temperature, pH and average depth (P < 0.05). *An. arabiensis*, *An. funestus* s.l and *An. demeilloni* showed zoophilic and exophilic tendencies. None of the anophelines tested for *P. falciparum* and *P. vivax* sporozoite infections were positive.

**Conclusion:**

In conclusion, malaria parasites and vectors existed in the highlands of Derashe District. Therefore, appropriate disease and vector control strategies must be designed and implemented to prevent potential outbreaks.

## Background

It has been generally known that malaria transmission usually only occurss in areas below 2,000 meters above sea level. However, this trend has now changed and has moved up to 2,500 meters above sea level, mainly due to climate change and land-use changes [1,2]. Moreover, there has been a spread of endemic malaria into the highland fringes, which are known to be non malaria endemic due to unsuitability of their low temperatures and relative humidity for anopheline development and reproductionon [3].

In most highland areas, communities are non-immune against malarial parasites [4]. Thus, a small increase in the abundance of vectors may lead to a significant malaria outbreak in the highlands. In addition, non-immune people living in highland areas frequently move to malaria endemic areas for various purposes such as searching for farm land and seeking job opportunities [5], which may pave the way for encroachment of the disease to the highland.

Epidemics have frequently been reported in the highlands of Ethiopia [6] and *An. arabiensis* was the species often reported from the highlands [7,8]. *An. christyi, An. demeilloni* and *An. coustani* are also other highland species in the country with no role in malaria transmission documented [7,9].

The highland region of Ethiopia with altitudes ranging between 1,500 and 2,500 meters above sea level are known to be prone to malaria epidemics [10]. This is mainly due to low endemicity of the disease in the higher altitudes [11] and the associated low protective immunity among the population. The prevalence of 3.2% was reported at an altitude range of 25, 00 to 3000 meters above sea level [12], which could partly be attributed to the expansion of the vectors into areas of higher altitudes. An increase in daily minimum temperature of 0.4°C per decade has been recorded in the highlands of Ethiopia [13]. Apparently, the rise in temperature favours the development of the malaria parasite in mosquitoes [11]. Expansion of these vectors to highland areas is a serious threat because most of the Ethiopian population lives in the highlands.

Nevertheless, there is limited information on malaria transmission in the highlands of Ethiopia and no report has been documented on the ecology and behaviour of malaria vectors in such regions. Therefore, the current study was aimed to determine the ecology, behaviour, species composition, distribution and other entomological indices of the vectors in the highland and lowland localities of Southern Ethiopia.

## Methods

### Study area

**Figure 1:**
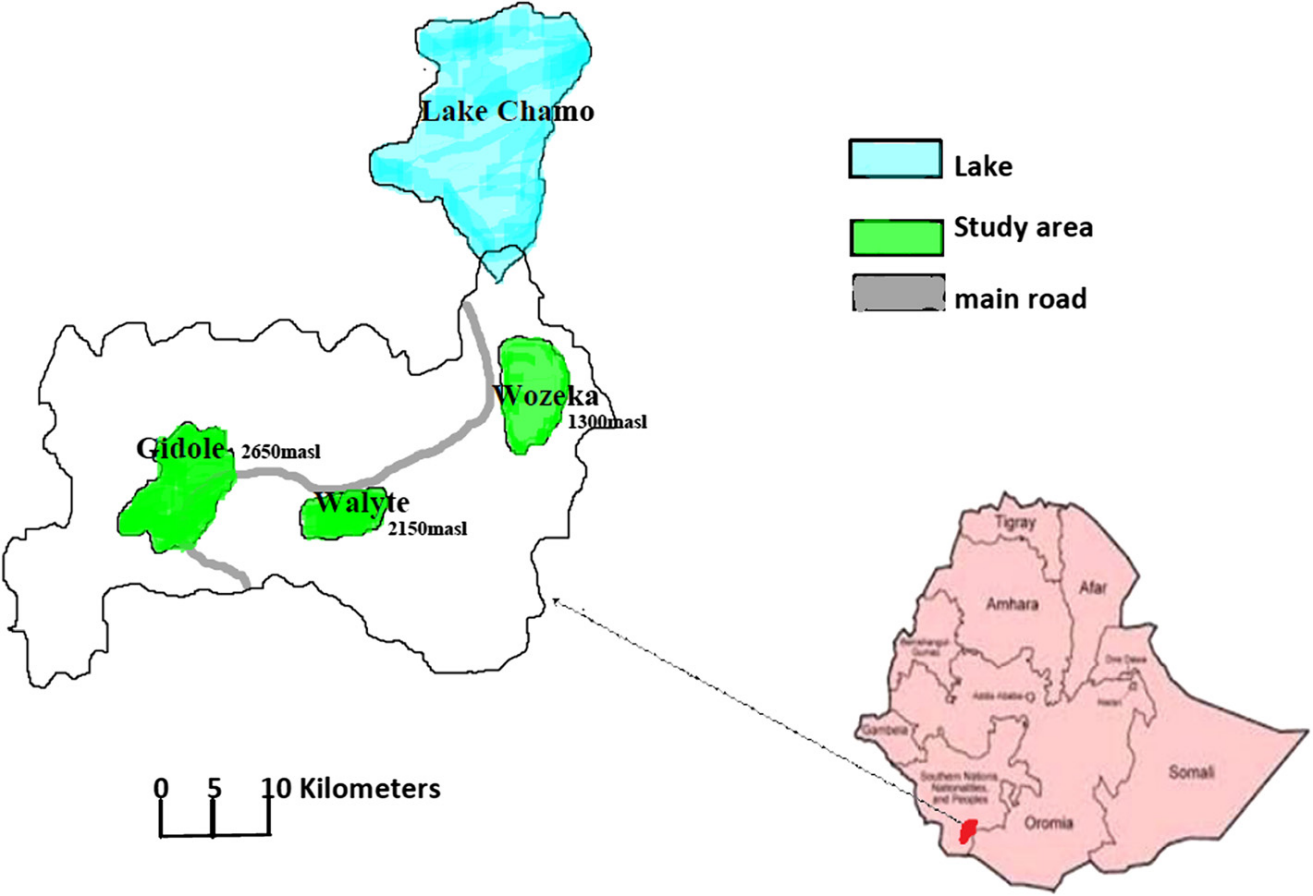
**Location of the study area in Ethiopia.**

The study was conducted in three rural localities in the southern part of Ethiopia with altitudes ranging between 1,300 and 2,650m above sea level. Mosquito sampling localities were Wozeka in the lowland (1300 masl), Walyte (2150 masl and 11 kms far from Wozeka) and Gidole town (2650 masl and 5 kms away from Walyte) in the highland (Figure 1). The area is rich in fertile soil and farming is the main mode of living. Sorghum, maize and teff are the main cereal crops that the local people produce. The rainy season is bi-modal in which the long rains occur from April to June and the short rains from September to October. The main malaria seasons in the area are following these rainy months i.e. July, August, November and December. The mean annual rainfall reaches up to 1600 millimeters and the mean annual temperature is 23°C. Water bodies such as streams and rivers commonly exist in the area. Malaria cases were reported several times from the highlands. As it is indicated in the first half year report of 2004 E.C., the overall malaria prevalence in the District was 4.2% and it had a prevalence of 6.4%, 2.2% and 2.6% in Wozeka (lowland), Walyte and Gidole (the two highland localities), respectively (Unpublished report from the District Health Office).

### Entomological survey

A monthly longitudinal entomological study was carried out from October 2011 to February 2012 in the three localities of Derashe District.

### Larval mosquito collection

Anopheline larvae were sampled from breeding habitats such as rain pools, irrigation canal, springs and streams, monthly between October 2011 and February 2012 by dipping using a standard dipper (11.5 cm diam and 350 ml capacity), pipettes, and white plastic pans [14]. When mosquito larvae were present, ten dips were taken at intervals along the edge [15]. Samplings were always done by the same individual in the morning (10:00–12:00 hrs) or afternoon (15:00–17:00 hrs). All third and fourth instar anopheline larvae collected were preserved in 2% formalin for later mosquito species identification [16]. Simultaneously with larval sampling, water temperature and water pH was measured using a Mercury Thermometer and pH Indicator respectively. Water depth was measured using a metal ruler at different points of each habitat and average depth was recorded. Water current was determined by visual inspection and categorized as slow flowing or still. Turbidity was estimated by taking water samples in glass test tubes and holding them against a white paper background to categorize them as either clear or turbid [17]. Intensity of light was visually categorized as sun-lit and shaded. The presence of aquatic vegetation and mats of algae was visually determined. Distance to the nearest house was measured by footsteps. This was then categorized into three classes (e.g. 1 = 0–100 m, 2 = 100–300 m, and 3 for distances >300 m) [17].

### Adult anopheline collections

A list of households of the three localities was used as a sampling frame, with an assumption of similar exposure of houses; a total of 33 households (11 from each locality) were systematically selected from houses that are found near mosquito breeding sites (in pits, artificial ponds, stream margins, springs and rain pools). The same houses were used throughout the study and when the occupants refused to allow use of their house, the household nearest to it was selected.

### Indoor resting collection

Space spray method [15] was performed early in the morning from 07:00 to 09:00 hours in five houses in each locality using aerosol (Roach killer, M/S Kafr EI Zayat, Egypt) and knocked down mosquitoes were collected. The mosquitoes were sorted out to their feeding stages as unfed, fresh fed, half gravid and gravid. After morphological identification to species, mosquitoes were preserved individually in Eppendorf tubes containing silica gel for later detection of sporozoite infections, blood meal sources and sibling species identifications in the laboratory (see below).

### Outdoor resting collection

Outdoor resting mosquitoes were collected from artificial pit shelters (1.5m of depth, 1.2m of length and 1m of width) using a sucking tube [18]. Two pit shelters were prepared in each locality in the vicinity of houses used for sampling mosquitoes with the other methods. The captured mosquitoes were treated in a similar manner as that of indoor resting collections.

### Overnight mosquito collection

Dry cell battery-operated CDC light traps (John W. Hock Company) [15] were placed inside houses near the bed of occupants who slept under insecticide untreated nets and were allowed to operate from 18:00 to 06:00 hours. In the mornings, mosquitoes were collected from each trap using an aspirator. These were transferred to test tubes and killed with chloroform [15] after which they were treated in a similar manner as described above.

### Species identification based on morphological features

Species identification of adult anopheline mosquitoes was performed using morphological characteristics of their palps, wings, abdomen and legs [19]. Larvae were also identified morphologically by mounting individually in gum-chloral on a microscope slide [19].

### Identification of sibling species by PCR

Sibling species of *An. gambiae* complex which is comprised of two species, *An. arabiensis,* and, *An. quadriannulatus* species B in Ethiopia were distinguished by Polymerase Chain Reaction (PCR) assay [20].

### Sporozoite infection determination

Sandwich Enzyme Linked Immuno-Sorbent Assay (ELISA) [21] was employed to determine sporozoite infection using the head and thorax of a mosquito.

### Blood meal source determination

Fresh fed mosquitoes were tested for the source of blood meal using a direct ELISA procedure described by Bier *et al*. [22].

### Ethical considerations

Consent was sought from each kebele administration, and informed consent was also sought from head of households from where mosquitoes were collected. The study obtained ethical clearance from the Institutional Review Board of ALIPB.

### Data analysis

The data obtained from the study was computerized using Epidata version 3.1 data entry format and analyzed by statistical software, STATA version 11. In addition to simple descriptive statistics, chi-square test, t-test, correlational analysis, analysis of variance (ANOVA) and non parametric Kwallis test were applied to determine associations and differences among different variables. All statistical tests and generalizations were done by assuming 95% confidence interval and 5% level of significance.

## Results

### Larval mosquito species composition and abundance

Six *Anopheles* species including *An. arabiensis, An. christyi*, *An. demeilloni, An. funestus, An. pharoensis* and *An. cinereus* were identified from larval collections. *An. demeilloni* was sampled from the three villages but it was more prevalent in Gidole. *An. christyi* was identified only from larval collection and existed in significantly high density in the highland locality (Gidole) (P < 0.05). Likewise, *An. cinereus* was found in Walyte and Gidole but it was scarce (Table 1).

**Table 1:** **Species composition and adult and larval densities of** ***Anopheles*** **in Wozeka (Lowland), Walyte and Gidole (Highland) (October 2011 to February 2012), Southern Ethiopia**

**Species**	**Wozeka n (larval density/10dips)**	**Walyte n (larval density/10dips)**	**Gidole n (larval density/10dips)**	**Total n (larval density/10dips)**
*An. christyi*	0 (2.7)	0 (0)	0 (3.7)	0 (3.5)
*An. arabiensis*	59 (3.1)	0 (0)	0 (3)	59 (3.1)
*An. demeilloni*	6 (1)	3 (0)	11 (3)	19 (3)
*An. cinereus*	0 (0)	1 (0)	0 (4.6)	1 (4.6)
*An. funestus* s.l	18 (0)	0 (0)	0 (0)	18 (0)
*An. pharoensis*	4(0)	0 (0)	0 (0)	4 (0)
Total	87 (2.9)	4 (3.4)	11 (3.4)	102 (3.3)

Along with larval collection, four kinds of breeding habitats were determined in Gidole and Wozeka, which included rain pools, streams, springs and irrigation canals. Despite the presence of streams and artificial ponds, no larvae were found in Walyte throughout the study period. Rain pools served as the main breeding habitats during the rainy months (October to December) in Gidole town. Pools from spring water and a small temporary stream which crosses the town created habitats during the dry months (January and February). Derek Wonz River and an irrigation canal were the two principal larval habitats in Wozeka during the dry months and no positive habitat was identified during the rainy months.

**Figure 2:**
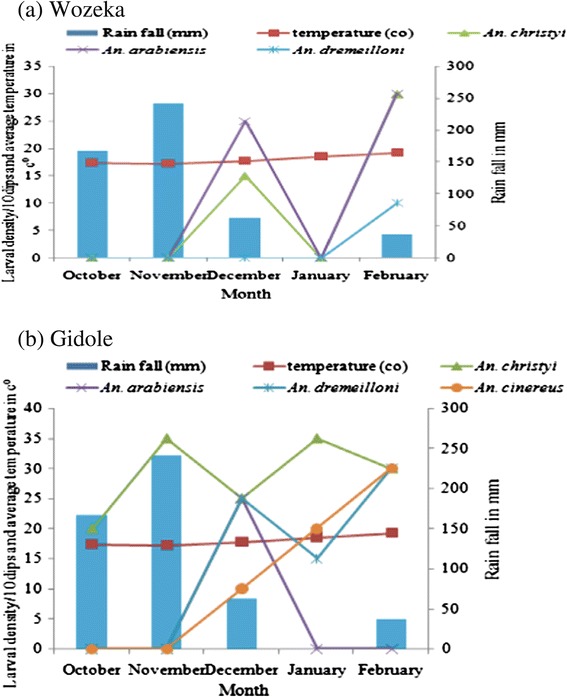
**Monthly rain fall, average temperature and larval densities of** ***Anopheles*** **species in Wozeka (lowland) (a) and Gidole (highland) (b) localities in southern Ethiopia.**

Larval density increased after the rain ceased in both highland and lowland localities. The maximum number of anopheline larvae were collected in January in Gidole and in February in Wozeka. *An. arabiensis*, the commonest species in the lowland was abundant in February while *An. christyi* existed only in December. The latter was collected more frequently in November and January from the highland. On the other hand, larval density of *An. demeilloni* and *An. cinereus* was relatively high in February (Figure 2).

Each species of *Anopheles* showed significant habitat preference (χ^2^ = 472.3, P < 0.05). *An. arabiensis* and *An. demeilloni* were sampled from pools and edges of streams, whereas *An. christyi* was predominantly collected from rain pools. *An. cinereus* on the other hand showed preference to water pools created from spring water. In addition, *An. christyi* and *An. arabiensis* coexisted in irrigation canals in the lowland area though their density was low (Table 2).

**Table 2:** **Larvae of** ***Anopheles*** **collected from various habitats in Wozeka and Gidole (October 2011 to February 2012), Southern Ethiopia**

**Breeding sites**		***An. christyi***	***An. arabiensis***	***An. demeilloni***	***An. cinereus***	**Total (%)**
Stream n (%)	Wozeka	26 (38.8)	141 (94)	1 (100)	0 (0)	168 (77.1)
	Gidole	3 (0.9)	0 (0)	106 (93)	0 (0)	109 (22)
Irrigation canal n (%)	Wozeka	41 (62.2)	9 (6)	0 (0)	0 (0)	50 (22.9)
	Gidole	0 (0)	0 (0)	0 (0)	0 (0)	0 (0)
Rain pool n (%)	Wozeka	0 (0)	0 (0)	0 (0)	0 (0)	0 (0)
	Gidole	223 (64.3)	27 (100)	4 (3.5)	1 (14.3)	255 (51.5)
Spring pool n (%)	Wozeka	0 (0)	0 (0)	0 (0)	0 (0)	0 (0)
	Gidole	121 (34.9)	0 (0)	4 (3.5)	6 (85.7)	131 (26.5)

Temperature was significantly and positively correlated with mean larval density of most *Anopheles* species identified both from highland and lowland (P < 0.05). More anopheline larvae were collected from shallow habitats having low pH in Gidole. Nevertheless, *An. arabiensis* was abundantly found in relatively deeper and warmer water habitats with high pH in Wozeka but its existence in a single habitat in the highland limited further analysis. Average water depth and pH were inversely related with *An. christyi* in the lowland but only average depth in the highland (Table 3).

**Table 3:** **Correlation coefficients between some environmental variables and densities of anopheline larvae from Wozeka and Gidole, Southern Ethiopia**

**Variables**	**Total**		***An. arabiensis***		***An. christyi***		***An. demeilloni***	
	**Wozeka**	**Gidole**	**Wozeka**	**Gidole**	**Wozeka**	**Gidole**	**Wozeka**	**Gidole**
Water temperature	0.97	0.14	0.99	Unidentified	0.99	−0.04	Unidentified	0.14
Water pH	0.18	−0.12	0.56		−0.46	0.04		−0.99
Water depth	0.12	−0.26	0.94		−0.46	−0.3		−0.096

Analysis indicated that larval habitats of *An. christyi* are characterized as shaded, still, turbid, permanent and contain natural water with vegetation and mats of algae. Similarly, *An. demeilloni* appear to survive in habitats with vegetation and mats of algae (Table 4). On the other hand, habitats of larvae of *An. arabiensis* are clear, sun-lit, permanent, still, without vegetation and situated close to human habitations. Larvae were totally absent in habitats having environmental characteristics other than those mentioned.

**Table 4:** **Characteristics of water habitats and mean densities of two common larval** ***Anopheles*** **species**

**Character**	**Variable**	***An. christyi***			***An. demeilloni***		
		**Mean ± SE**	**F**	**P**	**Mean ± SE**	**F**	**P**
Intensity of light	Sun-lit	2.9 ± 0.08	115.28	0	3 ± 0.35	0.5	0.2403
	Shaded	5.4 ± 0.08			3 ± 0.028		
Water current	Slow flowing	3 ± 0.105	42.61	0	3 ± 0	0.04	0.4252
	Still	4 ± 0.115			3 ± 0.036		
Turbidity	Turbid	3.6 ± 0.14	0.65	0	Unidentified		
	Clean	3.5 ± 0.09					
Vegetation	Present	3.9 ± 0.139	13.07	0.0002	3.5 ± 0.957	6.77	0.0052
	Absent	3.3 ± 0.097			3 ± 0.018		
Permanence	Permanent	4.4 ± 0.136	72.64	0	3.7 ± 0.667	21.42	0
	Temporary	3 ± 0.09			3 ± 0		
Distance to the nearest house	100m-300 m >300 m	3.5 ± 0.084 3.8 ± 0.322	1.20	0.1372	Unidentified		
Origin of habitat	Natural	4.3 ± 0.133	70.32	0	3 ± 0.036	0.04	0.4252
	Artificial	3 ± 0.091			3 ± 0		
Presence of algae	Present	4.5 ± 0.147	71.74	0	3.5 ± 0.957	6.77	0.0052
	Absent	3.1 ± 0.087			3 ± 0.018		

### Adult mosquito collection

Five Anopheles species including *An. arabiensis*, *An. demeilloni*, *An. funestus*, *An. pharoensis* and *An. cinereus* were identified from adult collections. Adults of *An. arabiensis* were sampled only from the lowland locality (Wozeka) and it outnumbered the rest of the mosquitoes. Its presence was confirmed from species specific PCR and it was the only member of *An. gambiae* found in the locality. Similarly, adults of *An. funestus* s.l and *An. pharoensis* were also collected in small numbers from the lowland (Table 1).

**Figure 3:**
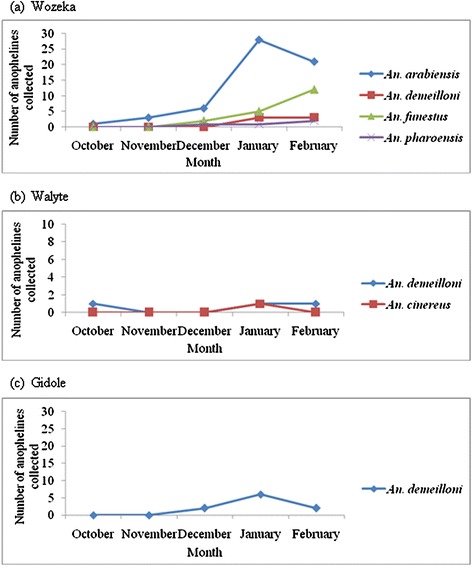
**Monthly adult** ***Anopheles*** **species densities in Wozeka (lowland) (a), Walyte (highland) (b) and Gidole (highland) (c) in Southern Ethiopia.**

The trend of monthly adult anopheline density followed a similar pattern as the larval density peaking in January (45.1%) shortly after the rainy season. The majority of *An. arabiensis* and *An. demeilloni* were collected in January while *An. funestus* s.l was collected in February from the lowland locality (Wozeka) (Figure 3). On the other hand, *An. pharoensis* showed inconsistent occurrence.

More than half of the adult anophelines were collected indoors by CDC light traps and space spray with the remainder collected outdoors from artificial pit shelters. *An. arabiensis* and *An. funestus* s.l were collected more frequently outdoors, whereas *An. demeilloni* was collected abundantly indoors, particularly in the highland locality (Gidole).

Comparable resting behaviour was noticed between *An. arabiensis* and *An. funestus* s.l, both being strongly exophilic. On the other hand, *An. demeilloni* showed mixed resting behaviour i.e. both endophilic and exophilic habit equally in the lowland. However, it became more endophilic as altitude increased (from 1300 masl in Wozeka to 2650 masl in Gidole), while its outdoor resting density significantly declined (χ^2^ = 11.7, P < 0.01). Anopheline density decreased from low lying Wozeka to the highland Gidole (P < 0.01). *An. arabiensis* had relatively high indoor density followed by *An. demeilloni* and *An. Pharoensis,* while *An. funestus* s.l was the species with the lowest indoor density. High indoor density was recorded in January particularly for *An. arabiensis* and *An. Demeilloni,* whereas in February the highest recording was for *An. funestus* s.l. Conversely, *An. pharoensis* showed inconsistent indoor density (Table 5).

**Table 5:** **Indoor and outdoor resting densities of four** ***Anopheles*** **species in the lowland (Wozeka) and highlands (Walyte and Gidole) of Derashe District (October 2011 to February 2012), Southern Ethiopia**

**Species**	**Indoor density**		**Indoor resting density**		**Outdoor resting density**	
	**Highland n/trap/night**	**Lowland n/trap/night**	**Highland n/day/hut**	**Lowland n/day/hut**	**Highland n/day/pit**	**Lowland n/day/pit**
*An. arabiensis*	0	0.18	0	0.7	0	2.1
*An. demeilloni*	0.06	0.03	1	1	0.33	1
*An. funestus* s.l	0	0.01	0	1	0	1.5
*An. pharoensis*	0	0.04	0	0	0	0

### *Plasmodium* Sporozoite infection rate

None of 96 anophelines tested for *P. falciparum* and *P. vivax* sporozoite infections was positive.

### Host preference

A total of 61 fresh fed anophelines were tested to detect the source of their blood meals. The majority of the blood meals of *An. Arabiensis*, 27(66%), *An. Demeilloni*, 4(67%) and *An. funestus* s.l., 9(64%) originated from bovine feeding, showing these species to be more zoophilic. A few samples had mixed blood meals, while the source of some others could not be identified in the highlands of Southern Ethiopia.

## Discussion

This study revealed the occurrence of *An. arabiensis*, *An. demeilloni*, *An. christyi, An. funestus* s.l, *An. pharoensis* and *An. cinereus* in the lowlands and highlands of Southern Ethiopia.

Investigation on characterization of breeding habitats showed that stream pools were the main breeding habitats of *An. arabiensis.* Similarly, the studies in Central and South-Central Ethiopia [23,24] showed the edges and beds of streams serve as breeding habitats for this vector during low rainfall season. On the other hand, *An. christyi* larvae were found in all kinds of habitats but abundantly in rain pools. Its ability to exist in such type of sites was reported from Central Ethiopia (Akaki) [7]. Similar to *An. arabiensis,* pools along streams serve as main breeding habitats for *An. demeilloni*.

Despite the presence of a number of streams and lower altitude, fewer numbers of anopheline larvae were collected from Wozeka as compared to Gidole. This might possibly be due to frequent drainage of the streams by the local people to their farms and directing back when not in use. This could disturb larvae. The absence of rain pools, swamps and springs which normally serve as potential breeding habitats during the rainy and dry months also played its role in this. Rain water usually dries up within a short period of time, which might be associated with the nature of the soil.

In spite of larval abundance in the highland Gidole, adults were rarely collected, this might be due to different biological, methodological and environmental factors. One could be the unsuitability of the low local temperature and humidity that hinder the complete development of larva to adult [4]. Another reason could be the low number of sampling sites and methods. Basically, mosquitoes disperse in all directions in search of host and resting place and as such it is difficult to capture abundantly when the area is wide and the sampling tools are small in number. In Gidole, there is dense vegetation all over the area which naturally can serve as resting sites, and hence, mosquitoes tend to avoid pit shelters.

Relatively high numbers of adults and larvae were collected in December, January and February. This may imply that these months could be among malaria risk times in the study area and it is a bit later than one of the two malaria transmission seasons in Ethiopia, which occurs from September to December and March to May [25]. The extended rain fall up to late November which later created favourable breeding habitats for the anophelines might have contributed to the prolongation of the transmission season.

The complete absence of *An. funestus* s.l larvae in the study area might have a connection with its peculiar preference for a large, permanent or semi-permanent body of fresh water with emergent vegetation, such as swamps, large ponds and lake edges [26] which are absent in the study localities. Adults of this species might have originated from inaccessible habitats such as solid surfaces, cracks in stone walls and stems of larger trees or it might be due to long flights from Lake Chamo which is 7 km far away from the study area. Adams [27] proved that mosquitoes could fly up to 8 Km with a prevailing breeze.

The study also showed the existence of *An. arabiensis* and *An. funestus* s.l in the lowland (Wozeka). Although sporozoite infections could not be detected from the former species, no other anopheline could be incriminated as a vector of malaria other than this species. While *An. arabiensis* plays a primary role as a major malaria vector in Ethiopia, *An. funestus* and *An. pharoensis* have been indicated as secondary vectors [28]. Similar to our finding, Taye *et al.* (2005) indicated the presence of these three malaria vectors in Sille of Southern Ethiopia, 40 Km from the present study area [29].

It was found that *An. demeilloni* was the principal highland *Anopheles* species in the study area. This is in agreement with the finding in Western Kenyan highlands where it was the second most abundant next to *An. christyi* [30]. Despite its scarcity*, An. cinereus* also showed highland existence both in its aquatic stage and adult; and its presence in highland areas has been reported previously [7]. Nevertheless, both species are not known to transmit malaria in Ethiopia and the rest of Africa.

We found that *An. arabiensis* showed zoophilic and an exophilic tendency, which is in harmony with what was documented before [21]. The preference of *An.arabiensis* to feed on cattle and rest outside after feeding in this study strengthens the findings of the study in Chalo of Southern Ethiopia, 65 km away from the current study area [31]. The experimental study in Tanzania also confirmed its tendency to escape from houses after feeding [32]. However, *An. funestus*, which is known to be endophilic and anthropophilic [33], exhibited exophilic and zoophilic behaviour. This might be related to variation in species as the *An. funestus* s.l group contains ten sibling species [30,33] with some showing inherent zoophilic habit. On the other hand, *An. demeilloni* showed entire zoophilic behaviour concordantly with what was reported from Kenya [30].

Variation in resting behaviour was noticed in *An. demeilloni* as altitude varied. It was shifted from exophilic in the lowland to endophilic in the highland. It is well known that temperature affects the biology of anophelines [34]. Therefore, they can avoid the effect of low temperature and unstable humidity in high altitudes by resting indoors as addressed in a study from the Cameroonian highlands [3].

Absence of *Plasmodium* sporozoite infection in the entire anophelines tested might be because of low prevalence of the sporozoite in the mosquito population in the study area or due to low numbers of anophelines collected.

A study in Kenya showed that certain areas in the highlands are more prone to malaria epidemics than others while the climate and altitude are similar [35]. This could be associated with terrain characteristics such as the shapes of the hills which determine the availability and stability of vector breeding habitats and subsequently the level of malaria transmission. Likewise, the lowest number of anophelines and the complete absence of larvae in Walyte might be due to the hilly nature of the locality which is devoid of suitable breading habitat during the rainy months. Despite the presence of streams which normally create breeding sites when the rain ceases, no anopheline larvae were recovered. This calls for further investigations.

## Conclusion

In conclusion, six *Anopheles* species were identified in the present study. *An. arabiensis* and *An. funestus* s.l were found to be important species in the lowland area. Despite lack of sporozoite infected *Anopheles* species*,* the former might be the major vector in the area as it is in the country. However, *An. Pharoensis,* which is a secondary vector in the country was scarce. In spite of the presence of malaria parasite and anophelines in the highlands of Derashe District, no appropriate disease and vector control strategies had been designed and applied before. In addition, the combined effect of the continuous movement of people in the area to lowlands in search of farm lands and job opportunities and the presence of few anophelines as well as low immunity may cause the occurrence of epidemics. Therefore, strategies considering vector resting and feeding behaviour should be designed and promptly employed for the control of both larval and adult mosquitoes.
